# Therapeutic potential of vasoactive intestinal peptide and its receptor VPAC2 in type 2 diabetes

**DOI:** 10.3389/fendo.2022.984198

**Published:** 2022-09-20

**Authors:** Xintong Hou, Dan Yang, Guimei Yang, Mengnan Li, Jian Zhang, Jiaxin Zhang, Yi Zhang, Yunfeng Liu

**Affiliations:** ^1^ Department of Endocrinology, First Hospital of Shanxi Medical University, Taiyuan, China; ^2^ First Clinical Medical College, Shanxi Medical University, Taiyuan, China; ^3^ Department of Pharmacology, Shanxi Medical University, Taiyuan, China

**Keywords:** vasoactive intestinal peptide, VPAC2, insulin secretion, glucose-dependence, VPAC2-selective agonists

## Abstract

Owing to the increasing prevalence of type 2 diabetes, the development of novel hypoglycemic drugs has become a research hotspot, with the ultimate goal of developing therapeutic drugs that stimulate glucose-induced insulin secretion without inducing hypoglycemia. Vasoactive intestinal peptide (VIP), a 28-amino-acid peptide, can stimulate glucose-dependent insulin secretion, particularly by binding to VPAC2 receptors. VIP also promotes islet β-cell proliferation through the forkhead box M1 pathway, but the specific molecular mechanism remains to be studied. The clinical application of VIP is limited because of its short half-life and wide distribution in the human body. Based on the binding properties of VIP and VPAC2 receptors, VPAC2-selective agonists have been developed to serve as novel hypoglycemic drugs. This review summarizes the physiological significance of VIP in glucose homeostasis and the potential therapeutic value of VPAC2-selective agonists in type 2 diabetes.

## Introduction

Diabetes is a chronic disease with multiple etiology and a complicated pathogenesis, with a global prevalence that is currently increasing annually. According to the statistics released by the International Diabetic Federation in 2021, 537 million people suffer from diabetes worldwide, with an estimated global prevalence of 10.5%. The number of cases is expected to reach 783 million in 2045, with the prevalence rising to 12.2% (1). Of this, 90% are patients with type 2 diabetes (2). Type 2 diabetes is a metabolic disease characterized by chronic hyperglycemia and insulin resistance. Chronic hyperglycemia leads to glucose toxicity to vital organs, including the eyes, kidneys, and nerves; therefore, maintenance of glucose homeostasis in the disease management of type 2 diabetes is crucial. Although multiple oral glucose-lowering drugs can effectively improve blood glucose levels in patients with type 2 diabetes, they may also cause hypoglycemia as a serious side effect because the glucose-lowering activity of these drugs is independent of the blood glucose levels of patients. If this side effect occurs frequently, it will lead to life-threatening cardiovascular and cerebrovascular complications. Currently, novel hypoglycemic drugs exhibit the unique, ideal property of promoting insulin secretion in a glucose-dependent manner without causing hypoglycemia (3). There is evidence that vasoactive intestinal peptide (VIP), a peptide hormone, promotes insulin secretion in this manner. Therefore, VIP may act as an insulinotropic drug without increasing the risk of hypoglycemia following administration. One of the cell receptors associated with VIP is VPAC2, which regulates insulin secretion; thus, this interaction may potentially be key towards a novel therapy. This review summarizes the physiological significance of VIP in glucose homeostasis and the therapeutic potential of VPAC2-selective agonists in the treatment of type 2 diabetes.

## Production of VIP and the associated receptors

VIP (a 28-amino-acid peptide) was first isolated from the duodenums of pigs in the early 1970s and was considered to be a gut hormone with vasodilatory effects (4). It was soon found to be widely distributed throughout the central nervous system and peripheral tissues (5, 6), including the brain, gastrointestinal tract, pancreas, immune organs and cardiovascular system (7, 8). Its distribution reveals its pleiotropic functions as a neurotransmitter, vasodilator, secretagogue and immunomodulator (9, 10). VIP is generated by the splicing and processing of its precursor from prepro-VIP, containing 170 amino acids (11), in addition to being processed into peptide histidine methionine in humans or peptide histidine isoleucine in other animals, which perform many common physiological roles (12, 13). VIP is a member of the secretin peptide family belonging to the same family as pituitary adenylate cyclase-activating polypeptide (PACAP) and glucagon and glucagon-like peptide-1 (GLP-1), which are coupled to specific G-protein-coupled receptors (GPCRs) on the cell surface (14, 15). VIP mainly relies on stimulating cyclic adenosine monophosphate (cAMP) production to play physiological roles in multiple tissues (16).

VIP exerts its physiological effects by binding to two receptor subtypes belonging to class B of GPCRs, namely VPAC1 and VPAC2 (17). PACAP can also activate the same receptors to exert functions, as its amino acid sequence shares 68% homology with that of VIP (18). PACAP has a specific receptor, termed PAC1, which exhibits a high affinity for it, whereas VIP and PACAP exhibit equally high affinity for VPAC1 and VPAC2 (19, 20). VIP receptors are expressed throughout the body and elicit a wide range of biological effects, such as relaxation of smooth muscles, promotion of gastrointestinal motility and regulation of hormone secretion (21). The two specific receptors for VIP are expressed on pancreatic islets (22), where VPAC1 is mainly responsible for glucagon secretion and hepatic glucose production (23, 24), while VPAC2 plays a role in improving glucose tolerance by stimulating insulin secretion. Additionally, VPAC2 appears to be less involved in the glycogenolytic pathways of the liver (22, 25). Based on these characteristics of VIP receptors, they can potentially be targeted for the treatment of type 2 diabetes.

## Endocrine function and signaling of VIP in islets

**Figure 1:**
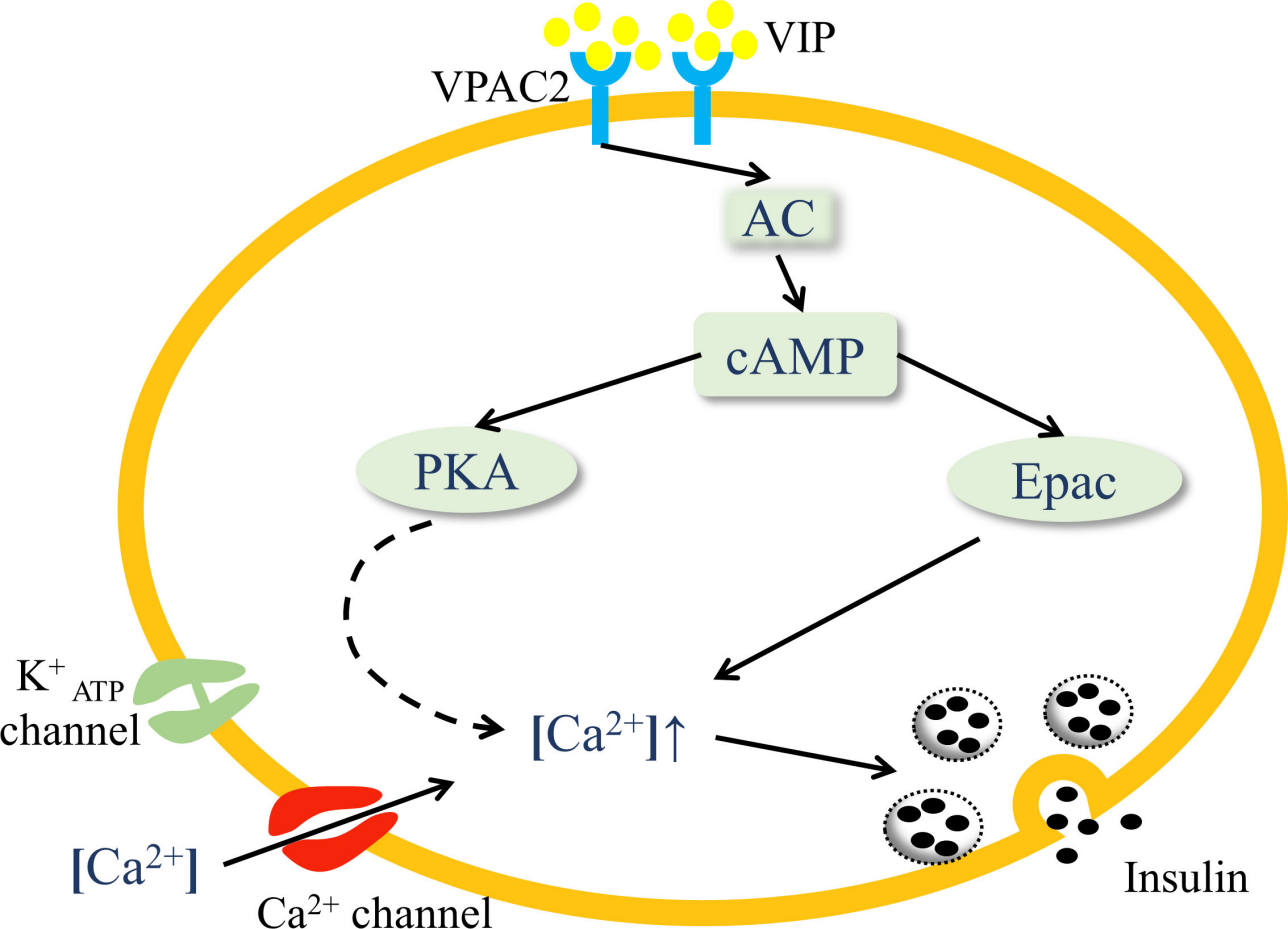
VIP activates the VPAC2 signaling pathway in pancreatic islets. VIP binding to VPAC2 receptors on β-cells activates AC and increases the concentration of cAMP, which activates PKA and the Epac family. PKA triggers the closure of ATP-dependent K^+^ channels, resulting in plasma membrane depolarization, and the opening of voltage-gated Ca^2+^ channels, which leads to an increased influx of Ca^2+^. Activation of Epac mobilizes the release of Ca^2+^ from internal storage. Both the processes cause elevated intracellular Ca^2+^ levels and the release of insulin through exocytosis.

Islets are innervated by parasympathetic, sympathetic, and sensory nerves (26). Immunohistochemistry has confirmed that VIP is localized in the postganglionic parasympathetic neurons, which originate from the dorsal motor nuclei of vagus nerves (27). VIP is released in islets after parasympathetic activation. VIP exerts two distinct endocrine functions in islets, namely glucagon secretion and insulin secretion, both of which are associated with glucose concentration (24). VIP-induced glucagon secretion occurs during hypoglycemia, while VIP exerts its role of promoting insulin secretion during hyperglycemia. In terms of glucose homeostasis, this review focuses on the function of VIP-stimulated insulin secretion. Glucose is the major factor triggering insulin secretion. Glucose breakdown increases ATP/ADP ratio in β-cells, closes ATP-dependent K^+^ channels, resulting in plasma membrane depolarization and the opening of voltage-gated L-type Ca^2+^ channels, which leads to an increased influx of Ca^2+^ to secrete insulin (28). The pathway of glucose-stimulated insulin secretion is modulated by other peptides, such as VIP, GLP-1 and PACAP. VIP amplifies the glucose-stimulated insulin secretion pathway through the cAMP cascade (29). VIP binding to VPAC2 receptors on β-cells preferentially interacts with G_s_ protein to activate adenylate cyclase (AC), resulting in a dose-dependent increase in cAMP (30, 31). As the second messenger, cAMP activates protein kinase A (PKA) and the Epac family of cAMP-regulated guanine nucleotide exchange factor (32, 33), both of which cause an increase in intracellular Ca^2+^ levels and induce insulin secretion (**Figure 1**) (22, 34). This mechanism is predominantly relevant in hyperglycemia, suggesting a glucose-dependent modality in the insulinotropic effect of VIP. The activity of other hormones to stimulate insulin secretion is also glucose-dependent, including GLP-1 and PACAP. VIP, GLP-1, and PACAP show equal effectiveness in stimulating insulin secretion (35). Interestingly, the combination of VIP and PACAP has no additive effect on the levels of insulin secretion, possibly because they share common receptors (36).

## Roles of VIP and VPAC2 in the pancreas

Several VIP-related animal models designed to study pancreatic islets are summarized here to demonstrate the role of VIP in the pancreas. In VIP-knockout mice, fasting insulin levels were found to be significantly elevated compared to those in wild-type mice. Moreover, their blood glucose levels in the VIP knockout mice did not decrease but instead increased (37), suggesting insulin resistance in VIP^-/-^ mice. No change in islet mass was observed in these mice. It was speculated that this observation may be due to the activity of other structurally similar peptides, such as PACAP, which can activate VIP-associated receptors to replace VIP effects in VIP^-/-^ mice (38). Yet, glucose abnormalities were seen in VIP^-/-^ mice, indicating that VIP was involved in the regulation of glucose homeostasis. In fact, mice overexpressing VIP effectively exhibited reduced blood glucose levels and elevated insulin levels. This effect was seen after feeding but not while fasting (39). *In vitro* studies, VIP secretion was found to correlate with glucose concentration. The islets isolated from mice overexpressing VIP were individually exposed to media containing different concentrations of glucose. A high glucose concentration significantly promoted VIP secretion in pancreatic islets and enhanced glucose-induced insulin secretion (39). Hypoglycemia has not yet been identified in these models, which means that VIP induces insulin secretion in a glucose-dependent manner. In addition, VIP-overexpressing mice were still able to maintain glucose tolerance after removing 70% of the pancreas (39).

As mentioned above, when VIP binds to VPAC2 receptors, insulin secretion is induced. Therefore, VPAC2 receptor knockout mice represent an important model to evaluate the role of VPAC2 in pancreatic islets. In the oral glucose tolerance test, VPAC2-deficient mice showed a similar glycemic response to wild-type mice, but insulin levels were significantly reduced in VPAC2-deficient mice (40). This suggests that the insulin sensitivity of VPAC2-deficient mice was markedly increased. This conjecture was confirmed in the insulin tolerance test. After insulin administration, blood glucose levels in VPAC2-deficient mice decreased to a greater extent than those in wild-type mice (40). These observations indicate that VPAC2 plays an important role in glucose homeostasis.

Previous studies have found that the effects of VIP on the pancreas are influenced by age and metabolic status (41). Islets from obese and lean mice of different ages were used to study the effect of VIP on insulin release. The results showed that VIP significantly enhanced insulin release in islets isolated from young obese mice, while islets from lean mice showed a very low sensitivity to VIP. In much older mice, VIP had no effect on insulin release, regardless of the sizes of the animals (obese or lean) (42). Therefore, age and metabolic status should be considered when discussing the effects of VIP on optimal insulin secretion (41).

## Roles of VIP in the liver-to-pancreatic neuronal relay

**Figure 2:**
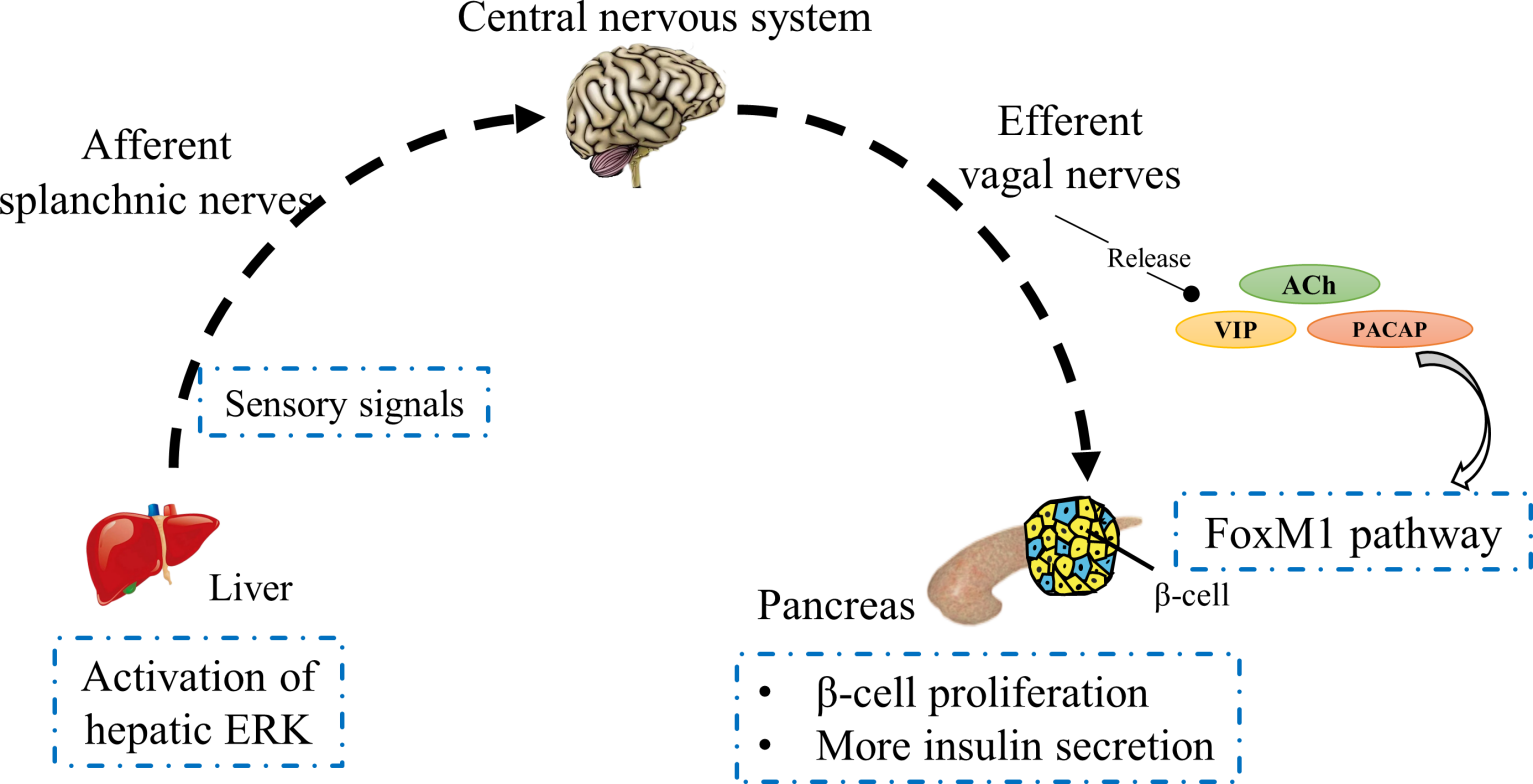
Schematic of a liver–brain–pancreas neuronal relay. In insulin-resistant individuals, hepatic ERK activation transmits sensory signals to the central nervous system through the afferent splanchnic nerves, which in turn reach the pancreas *via* vagal nerves. Vagal factors induce islet β-cell proliferation to secrete more insulin through activation of the FoxM1 pathway.

Islet β-cell mass contributes to glucose homeostasis by inducing compensatory responses based on physiological requirements of insulin (43, 44). In an insulin-resistant environment, such as that caused by obesity, neuronal signaling from the liver is involved in the compensatory proliferation of islet β-cells (45). Imai et al. has proposed that the activation of hepatic extracellular-signal regulated kinase (ERK) is transmitted to the central nervous system by afferent splanchnic nerves and then to the pancreas *via* efferent vagal nerves to promote β-cell proliferation (46); this hypothesis has been verified in different mouse models (47). Disruption of any portion of neuronal transmission from the liver to the pancreas inhibits β-cell proliferation, including inhibition of ERK phosphorylation, pharmacological deafferentation of splanchnic nerves, midbrain transection, and pancreatic vagotomy, which demonstrates the role of a liver–brain–pancreas neuronal relay in islet β-cell proliferation. Although it has been shown that hepatic ERK activation triggers neuronal transmission from the liver to the pancreas, how its ERK signaling travels down *via* visceral nerves is unknown (46). In this neuronal transmission, vagal nerve signals activate the forkhead box M1 (FoxM1) pathway in β-cells, thereby promoting compensatory proliferation of β-cells and augmenting insulin secretion (**Figure 2**) (48). FoxM1 is a key transcription factor for cell proliferation (49, 50). It has been reported that vagal signals activate the upregulation of FoxM1-related genes in islets, including *Foxm1*, cyclin-dependent kinase 1 (*Cdk1*), cyclin A (*Ccna*) and polo-like kinase 1 (*Plk1*), as well as *Mki67*, all of which are involved in the cell cycle and induce β-cell proliferation (47).

To demonstrate the role of vagal signals in the liver–brain–pancreas neuronal relay, mouse islets were treated with a combination of neurotransmitters released from vagus nerves, including acetylcholine (ACh), VIP and PACAP (26); the results showed that they indeed promoted β-cell proliferation. Additionally, gene expression analysis showed that the expression levels of FoxM1-related genes and *Mki67* gene were significantly increased (47). To explore the role of each of these neurotransmitters in the FoxM1 pathway, they were used individually to treat rat isolated islets, the above outcome was not observed. Meanwhile, when ACh was removed from the combined treatment of multiple neurotransmitters, no upregulation of these genes was observed in pancreatic islets, suggesting that ACh plays a decisive role in the FoxM1 pathway. Upon combined treatment of islets with ACh plus VIP or PACAP, the expression levels of FoxM1-related genes and *Mki67* gene were markedly increased, and the gene expression levels of ACh combined with VIP and ACh combined with PACAP were similar, causing significant proliferation of β-cells (47). ACh stimulates insulin secretion by activating G_q_ signaling (51, 52), while VIP and PACAP, as G_s_-signaling activators, exert insulinotropic effects (30), implying that vagal signals may activate the FoxM1 mechanism by simultaneously stimulating multiple pathways to enhance β-cell proliferation. At present, the exact molecular mechanism of vagal factors and the FoxM1 pathway is unclear and needs to be further investigated. It has previously been demonstrated that the content of VIP in the pancreas of diabetic mice is increased, but its effect on stimulating insulin secretion is lower than that in normal mice (53). One plausible reason is a diminished VIP-receptor sensitivity in diabetic mice, and another can be attributed to the inhibition of FoxM1 pathway activation. Recently, it has been found that the reduced expression levels of ACh in diabetic mice with poor islet function are associated with pancreatic autonomic nerve damage (54, 55), which may attenuate the role of vagal signals in activating the FoxM1 pathway, indirectly suggesting that neuronal signaling from the liver to the pancreas is impaired in diabetic mice. This may imply that patients with type 2 diabetes have a disorder in the liver–brain–pancreas neuronal relay. Elucidation of the mechanism governing vagal signals in neuronal transmission from the liver to the pancreas could open up new treatment avenues for type 2 diabetes.

## Development of VPAC2-selective agonists

The amino acid sequence of VIP is highly conserved in different species. The sequence of VIP is identical in mammals, except for the VIP sequence of guinea pigs, wherein 4 amino acids are replaced (56, 57). Amino acid substitutions also exist in some vertebrates such as chickens and frogs (58, 59), but this does not affect its biological activity. PACAP, the homolog of VIP, exists in two forms, specifically as a 38-amino acid peptide (PACAP38) and 27-amino acid peptide (PACAP27), with PACAP38 being its main form that plays a role in various tissues and organs (14). PACAP is one of the most conserved peptides in the secretin peptide family (60), with a consistent amino acid sequence in most animals except for one amino acid difference in chickens and frogs (15). The high sequence conservation of VIP and PACAP makes them attractive targets for disease treatment. Due to their structural similarity to GLP-1, VIP and PACAP are rapidly recognized and degraded by dipeptidyl peptidase-IV *in vivo*, contributing to their short half-lives, which results in transient effects (61). Although VIP and PACAP have been shown to induce insulin secretion, they can cause undesired redundant responses because of the wide distribution of their receptors in the body. These disadvantages constitute a technical barrier to the development of therapies for type 2 diabetes. Therefore, novel drug discovery and development of VIP analogues as well as a suitable drug delivery system are the main goals in developing VIP as hypoglycemic drugs.

Previous studies have demonstrated that the activation of VPAC2 receptors on β-cells is involved in promoting insulin secretion without triggering hepatic glycogenolysis and glucagon secretion, which results in hypoglycemic effects. Because of this, VPAC2 receptors can be a novel target for the treatment of type 2 diabetes. BAY55-9837 has been developed as a selective agonist for VPAC2, which was designed *via* site-directed mutations of VIP and PACAP (15, 62). It is a complete agonist for VPAC2 receptors and stimulates insulin secretion in a glucose-dependent manner. Islets isolated from rats and humans were placed into a medium at different glucose concentrations and supplemented with an appropriate amount of BAY55-9837. BAY55-9837 induced insulin secretion in islets in a medium containing 8 mmol/L glucose. However, its effect on insulin secretion was not observed in the 3 mmol/L glucose medium (62), effectively demonstrating that BAY55-9837 stimulates glucose-dependent insulin secretion. When BAY55-9837 was administered to rats either intravenously or subcutaneously, both the administration methods effectively induced insulin secretion and reduced blood glucose levels, and the hypoglycemic effect exhibited was similar to that of GLP-1. Additionally, there was no sign of hypoglycemia even with long-term continuous administration. Continuous subcutaneous injection of BAY55-9837 into rats resulted in a dose-dependent decrease in mean arterial pressure (62), which drew our attention. Unfortunately, BAY55-9837, similarly to natural VIP, exhibits a high sensitivity to dipeptidyl peptidase-IV, has poor metabolic stability (63), and deamidation at its asparagine sites results in rapid renal clearance (64). Therefore, this agonist would require continuous administration to maintain its pharmacological effectiveness, which limits its potential application in the treatment of type 2 diabetes.

To improve the stability of peptides, the recombinant peptide DBAYL (32 amino acids) was designed and produced using the gene recombination technology (65). The poor stability of BAY55-9837 is due to the high sensitivity of the H-S sequence at the N-terminus to dipeptidyl peptidase-IV, which prevents it from activating VPAC2 (61, 65). DBAYL adds a methionine to the N-terminus, blocking the N-terminal sensitive sequence, thus improving its stability and biological activity. DBAYL, a derivative of PACAP, activates VPAC2 to stimulate insulin secretion in a glucose-dependent manner, thereby effectively increasing glucose disposal (65). The accumulation of cAMP reflects the receptor potency of DBAYL. Its binding affinity to human VPAC1 receptors is only 1/1083 of its affinity to human VPAC2, and it has no activity against human PAC1 receptors. The half-life of DBAYL in mice is 1.98 h, which is approximately 23.8 folds that of BAY55-9837 *in vitro* (65, 66), prolonging the hypoglycemic time. In 3T3-L1 adipocytes treated with DBAYL, the expression of insulin receptor substrate 1 (IRS-1) and glucose transporter 4 (GLUT4) is significantly increased (65). IRS-1 is a key molecule for insulin signal transduction, and GLUT4 is an important carrier in glucose transport (67, 68). In addition, DBAYL increases the translocation of GLUT4, which is translocated from the cytoplasm to the cytomembrane in a non-insulin-dependent manner (65, 69). These proteins facilitate effective glucose uptake and utilization. Although DBAYL has some advantages, its half-life limits its use as a viable therapeutic drug for type 2 diabetes.

One approach to improve the stability of peptides is to attach polyethylene glycol (PEG) to peptides to prolong their action time. PEGylation increases the molecular weight of peptides, reduces renal clearance, prevents the degradation by peptidases, and maintains effective plasma concentration (70, 71). Therefore, the structure of BAY55-9837, which is prone to deamidation, was modified by adding a cysteine to the C-terminus to link it to PEG to produce its analogues, such as BAY (Q9Q28C32) PEG22 and BAY (Q9Q28C32) PEG43 (22- and 43-kDa PEGylated peptides, respectively) (72, 73). Both of them retained a high selectivity for VPAC2, extending their bioactivity. An intraperitoneal glucose tolerance test was performed in rats 3 h after subcutaneous injection of BAY (Q9Q28C32) PEG22 and BAY (Q9Q28C32) PEG43; both of these analogues induced substantially enhanced glucose disposal in rats. Moreover, a glucose tolerance test conducted 6 h later showed that only BAY (Q9Q28C32) PEG43 triggered a hypoglycemic effect. This indicates that the hypoglycemic action duration of BAY (Q9Q28C32) PEG22 and BAY (Q9Q28C32) PEG43 is at least 3 and 6 h, respectively (72). Compared with that of the original analogue BAY55-9837, these PEGylated peptides have significantly prolonged the half-lives. The structural stability of VPAC2-selective agonists remains to be improved, but it has been shown that the activation of VPAC2 on β-cells promotes insulin secretion in a glucose-dependent manner, which will inform future hypoglycemic drug innovations.

## Application of nanoparticles on VPAC2-selective agonists

Amino acid site-directed mutagenesis and PEGylation can prolong the half-life of BAY55-9837, but the duration of its hypoglycemic effect is currently not sufficient to allow its use as a viable treatment for type 2 diabetes. The emerging nanoparticle technology can be used to create applicable drug delivery systems that may be able to prolong drug action time and reduce side effects (74); hence, the current advances in the development of nanoparticle-based VPAC2-selective agonists are considered in this review. Nanoparticles, as sustained-release carriers, can slowly release VPAC2-selective agonists to maintain their effective concentration in patients and continually stimulate insulin secretion without causing hypotension and other potential side effects caused by current drug candidates, which are related to infusion rate and high dose (75, 76). Nanoparticle conjugation with VPAC2-selective agonists can not only prolong the half-lives but can also facilitate a targeted drug delivery to improve drug efficacy (74, 77). Selenium nanoparticles (SeNPs) have been proven to delay the progression of diabetes owing to their antioxidant properties (78, 79). However, the unstable state of selenium can easily cause its transformation, this can be prevented by modifying SeNPs with chitosan to maintain its stability; additionally, chitosan is also biocompatible (80–82). Therefore, the developed VPAC2-selective agonists, including BAY55-9837 and DBAYL, have been inserted into chitosan-modified SeNPs (CS-SeNPs) to form BAY-CS-SeNPs and SeNPs-CTS-DBAYL, respectively, which are regarded as potential drugs for the treatment of type 2 diabetes (66, 83). BAY-CS-SeNPs exhibited a reduced renal clearance due to its increased molecular weight but showed a significantly extended half-life of approximately 20.81 h. *In vitro* medium, a rapid release of BAY55-9837 was observed in the first 12 h of BAY-CS-SeNPs administration, which stimulated insulin secretion in a glucose-dependent manner; the drug release gradually slowed down until completion at 72 h. The early rapid release phase may meet the high insulin requirement for maintaining postprandial glucose, while the later slow release may maintain nocturnal insulin levels (83). Taken together, the release rate of BAY-CS-SeNPs satisfies the time curve of human physiological demand for insulin. Similarly, SeNPs-CTS-DBAYL was able to rapidly release DBAYL during the first 12 h, followed by a slow release up to 48 h (66). Its release rate, similarly to that of BAY-CS-SeNPs, fulfils human physiological requirement for insulin. The half-life of SeNPs-CTS-DBAYL in mice was up to 14.12 h, 7.1-fold that of DBAYL. The DBAYL released from SeNPs-CTS-DBAYL was able to highly and selectively activate VPAC2, induce glucose-dependent insulin secretion, and increase insulin receptor expression and glucose uptake. The treatment of hyperglycemia using SeNPs-CTS-DBAYL was evidently better than that using DBAYL. It inhibited oxidative damage in INS-1 cells due to the presence of CS-SeNPs. Diabetic mice with regular SeNPs-CTS-DBAYL administration for long periods of time exhibited regulated blood glucose levels, improved insulin sensitivity and lipid profile, and preservation of the normal morphology of pancreas and adipose tissues (66).

The widespread distribution of VIP and its receptors is a major obstacle to the development of hypoglycemic drugs. Studies have found that exosomes are nanoscale vesicles secreted by a variety of cells and have a low immunogenicity and a high drug-carrying capacity (84, 85). Exosomes can serve as ideal sustained-release carriers that are highly stable and thus can prolong the half-lives of drugs. Currently, exosomes are being used in the treatment of tumors and have yielded promising results (86); hence, attempts to adapt them in the development of VPAC2-selective agonists are underway. Because exosomes lack the ability to target pancreatic islets, they were combined with superparamagnetic iron oxide nanoparticles (SPIONs), which have the ability to target a specific location in the body to produce the desired therapeutic efficacy. SPIONs are biocompatible and can be targeted to transport drugs *via* an external magnetic force to the target location, thus reducing side effects (87). Recently, a new nanomedicine for VPAC2-selective agonists has been devised. It consists of BAY55-9837 loaded into SPIONs-modified exosomes named BAY-exosome-SPION (88). BAY-exosome-SPION targets and aggregates on β-cell surface under the action of a magnetic force, and the released BAY55-9837 binds to VPAC2 receptors on β-cells and enhances insulin secretion. During the release curve of the BAY-exosome-SPION, it was observed that BAY55-9837 was released rapidly during the first 5 h and continued to be released until 60 h later. Due to the presence of exosome-SPION, the half-life of BAY55-9837 in circulation was extended to 8.39 h and the plasma clearance rate was reduced (88), implying that the rapid degradation of BAY55-9837 was prevented, thereby reducing the frequency of administration. Diabetic mice were treated with BAY-exosome-SPION twice a day, and they showed significantly reduced glycosylated hemoglobin levels and body weights as well as improved lipid profiles after 8 weeks. Furthermore, no significant toxic damage was observed in mice treated with BAY-exosome-SPION, which indicated that BAY-exosome-SPION demonstrated a good biosafety profile *in vivo* (88). These nanomedicines exert ideal hypoglycemic effects through suitable drug delivery systems and possess unique advantages, paving new ways to improve VPAC2-selective agonists. The characteristics of the VPAC2-selective agonists mentioned above are summarized in **Table 1**. The developed VPAC2-selective agonists are currently in the early stages of *in vitro* and *in vivo* studies, and the clinical specificity and efficacy still need further research; hence, it would be a while before viable applications of these agents could be implemented in type 2 diabetes treatment.

**Table 1:** Structure and half-life of VIP and VPAC2-selective agonists.

Peptide	Structure	Half-life
VIP	HSDAVFTDNYTRLRKQMAVKKYLNSILN	Less than 1 min
PACAP27	HSDGIFTDSYSRYRKQMAVKKYLAAVL	5-10 min
PACAP38	HSDGIFTDSYSRYRKQMAVKKYLAAVLGKRYKQRVKNK	5-10 min
BAY55-9837	HSDAVFTDNYTRLRKQVAAKKYLQSIKNKRY	5 min
DBAYL	MHSDAVFTDQYTRLRKQLAAKKYLQSLKQKRY	1.98 h
BAY(Q9Q28C32)PEG22	HSDAVFTDQYTRLRKQVAAKKYLQSIKQKRYC-PEG22 kDa	–
BAY(Q9Q28C32)PEG43	HSDAVFTDQYTRLRKQVAAKKYLQSIKQKRYC-PEG43 kDa	3.5 h
BAY-CS-SeNPs	BAY55-9837, SeNPs and chitosan	20.81 h
SeNPs-CTS-DBAYL	DBAYL, SeNPs and chitosan	14.12 h
BAY-exosome-SPION	BAY55-9837, SPION and exosome	8.39 h

## Conclusion

Previous hypoglycemic drug and exogenous insulin administration reduces blood glucose levels regardless of the blood glucose levels *in vivo*, greatly increasing the risk of hypoglycemia. The emergence of novel hypoglycemic drugs circumvents this drawback. Several studies have demonstrated that the specific binding of VIP with VPAC2 receptors on β-cells can stimulate insulin secretion in a glucose-dependent manner, eliminating the risk of hypoglycemia. This characteristic has been used to design and produce VPAC2-selective agonists. In addition, VIP promotes β-cell proliferation through synergistic activation of the FoxM1 pathway in a liver–brain–pancreas neuronal relay, but the molecular mechanisms underlying this pathway still require further research. Currently, VPAC2-selective agonists are constantly being innovated to improve their stability and efficacy, which indicates the possibility of their successful clinical applications in type 2 diabetes treatment in the future.

## Author contributions

XH mainly wrote and revised the manuscript, and constructed the framework of the manuscript. ML and JZ provided constructive opinions on the formation of the manuscript. DY, GY and JXZ participated in the drawing of manuscript pictures and the investigation and sorting of documents. YZ and YL participated in topic design, manuscript writing, manuscript editing and providing instructional support. All authors contributed to the article and approved the submitted version.

## Funding

This work was supported by the National Natural Science Foundation of China (No. 81770776, 82073909 and 81973378), Research Project Supported by Shanxi Scholarship Council of China (No. 2020-172).

## Acknowledgments

We thank the National Natural Science Foundation of China (No. 81770776, 82073909 and 81973378), Research Project Supported by Shanxi Scholarship Council of China (No. 2020-172).

## Conflict of interest

The authors declare that the research was conducted in the absence of any commercial or financial relationships that could be construed as a potential conflict of interest.

## Publisher’s note

All claims expressed in this article are solely those of the authors and do not necessarily represent those of their affiliated organizations, or those of the publisher, the editors and the reviewers. Any product that may be evaluated in this article, or claim that may be made by its manufacturer, is not guaranteed or endorsed by the publisher.

## Abbreviations

AC, adenylate cyclase; ACh, acetylcholine; cAMP, cyclic adenosine monophosphate; *Ccna*, cyclin A; *Cdk1*, cyclin-dependent kinase 1; CS-SeNPs, chitosan-modified SeNPs; ERK, extracellular-signal regulated kinase; FoxM1, forkhead box M1; GLP-1, glucagon-like peptide-1; GLUT4, glucose transporter 4; GPCR, G-protein-coupled receptor; IRS-1, insulin receptor substrate 1; PAC1, PACAP receptor type 1; PACAP, pituitary adenylate cyclase activating polypeptide; PEG, polyethylene glycol; PKA, protein kinase A; *Plk1*, polo-like kinase 1; SeNPs, selenium nanoparticles; SPIONs, superparamagnetic iron oxide nanoparticles; VIP, vasoactive intestinal peptide; VPAC1, VIP/PACAP receptor type 1; VPAC2, VIP/PACAP receptor type 2.

## References

[1] Sun H Saeedi P Karuranga S Pinkepank M Ogurtsova K Duncan BB. IDF diabetes atlas: Global, regional and country-level diabetes prevalence estimates for 2021 and projections for 2045. Diabetes Res Clin Pract (2022) 183:109119. doi: 10.1016/j.diabres.2021.109119 34879977 PMC11057359

[2] Saeedi P Petersohn I Salpea P Malanda B Karuranga S Unwin N. Global and regional diabetes prevalence estimates for 2019 and projections for 2030 and 2045: Results from the international diabetes federation diabetes atlas, 9(th) edition. Diabetes Res Clin Pract (2019) 157:107843. doi: 10.1016/j.diabres.2019.107843 31518657

[3] Morales J. The pharmacologic basis for clinical differences among GLP-1 receptor agonists and DPP-4 inhibitors. Postgrad Med (2011) 123(6):189–201. doi: 10.3810/pgm.2011.11.2508 22104467

[4] Said SI Mutt V. Polypeptide with broad biological activity: isolation from small intestine. Science (1970) 169(3951):1217–8. doi: 10.1126/science.169.3951.1217 5450698

[5] Harmar AJ Fahrenkrug J Gozes I Laburthe M May V Pisegna JR. Pharmacology and functions of receptors for vasoactive intestinal peptide and pituitary adenylate cyclase-activating polypeptide: IUPHAR review 1. Br J Pharmacol (2012) 166(1):4–17. doi: 10.1111/j.1476-5381.2012.01871.x 22289055 PMC3415633

[6] Moody TW Jensen RT. Pituitary adenylate cyclase-activating polypeptide/vasoactive intestinal peptide [Part 1]: Biology, pharmacology, and new insights into their cellular basis of action/signaling which are providing new therapeutic targets. Curr Opin Endocrinol Diabetes Obes (2021) 28(2):198–205. doi: 10.1097/MED.0000000000000617 33449573 PMC7957349

[7] Reubi JC. *In vitro* evaluation of VIP/PACAP receptors in healthy and diseased human tissues. Clin Implications Ann N Y Acad Sci (2000) 921:1–25. doi: 10.1111/j.1749-6632.2000.tb06946.x 11193811

[8] Harmar AJ Sheward WJ Morrison CF Waser B Gugger M Reubi JC. Distribution of the VPAC2 receptor in peripheral tissues of the mouse. Endocrinology (2004) 145(3):1203–10. doi: 10.1210/en.2003-1058 14617572

[9] Delgado M Ganea D. Vasoactive intestinal peptide: a neuropeptide with pleiotropic immune functions. Amino Acids (2013) 45(1):25–39. doi: 10.1007/s00726-011-1184-8 22139413 PMC3883350

[10] Fahrenkrug J Hannibal J. Neurotransmitters co-existing with VIP or PACAP. Peptides (2004) 25(3):393–401. doi: 10.1016/j.peptides.2004.01.010 15134862

[11] Delgado M Pozo D Ganea D. The significance of vasoactive intestinal peptide in immunomodulation. Pharmacol Rev (2004) 56(2):249–90. doi: 10.1124/pr.56.2.7 15169929

[12] Itoh N Obata K Yanaihara N Okamoto H. Human preprovasoactive intestinal polypeptide contains a novel PHI-27-like peptide, PHM-27. Nature (1983) 304(5926):547–9. doi: 10.1038/304547a0 6571696

[13] Nishizawa M Hayakawa Y Yanaihara N Okamoto H. Nucleotide sequence divergence and functional constraint in VIP precursor mRNA evolution between human and rat. FEBS Lett (1985) 183(1):55–9. doi: 10.1016/0014-5793(85)80953-4 3838518

[14] Arimura A. Perspectives on pituitary adenylate cyclase activating polypeptide (PACAP) in the neuroendocrine, endocrine, and nervous systems. Jpn J Physiol (1998) 48(5):301–31. doi: 10.2170/jjphysiol.48.301 9852340

[15] Dickson L Finlayson K. VPAC and PAC receptors: From ligands to function. Pharmacol Ther (2009) 121(3):294–316. doi: 10.1016/j.pharmthera.2008.11.006 19109992

[16] Desbuguois B Laudat MH Laudat P. Vasoactive intestinal polypeptide and glucagon: stimulation of adenylate cyclase activity *via* distinct receptors in liver and fat cell membranes. Biochem Biophys Res Commun (1973) 53(4):1187–94. doi: 10.1016/0006-291x(73)90590-1 4356054

[17] Iwasaki M Akiba Y Kaunitz JD. Recent advances in vasoactive intestinal peptide physiology and pathophysiology: Focus on the gastrointestinal system. F1000Res (2019) 8:F1000 Faculty Rev-1629. doi: 10.12688/f1000research.18039.1 PMC6743256 31559013

[18] Onoue S Misaka S Yamada S. Structure-activity relationship of vasoactive intestinal peptide (VIP): potent agonists and potential clinical applications. Naunyn Schmiedebergs Arch Pharmacol (2008) 377(4-6):579–90. doi: 10.1007/s00210-007-0232-0 18172612

[19] Pisegna JR Wank SA. Molecular cloning and functional expression of the pituitary adenylate cyclase-activating polypeptide type I receptor. Proc Natl Acad Sci USA (1993) 90(13):6345–9. doi: 10.1073/pnas.90.13.6345 8392197 PMC46925

[20] Vaudry D Falluel-Morel A Bourgault S Basille M Burel D Wurtz O. Pituitary adenylate cyclase-activating polypeptide and its receptors: 20 years after the discovery. Pharmacol Rev (2009) 61(3):283–357. doi: 10.1124/pr.109.001370 19805477

[21] Laburthe M Couvineau A Marie JC. VPAC receptors for VIP and PACAP. Recept Channels (2002) 8(3-4):137–53. doi: 10.1080/10606820213680 12529932

[22] Winzell MS Ahren B. Role of VIP and PACAP in islet function. Peptides (2007) 28(9):1805–13. doi: 10.1016/j.peptides.2007.04.024 17559974

[23] Fabricius D Karacay B Shutt D Leverich W Schafer B Takle E. Characterization of intestinal and pancreatic dysfunction in VPAC1-null mutant mouse. Pancreas (2011) 40(6):861–71. doi: 10.1097/MPA.0b013e318214c783 21697765

[24] Bertrand G Puech R Maisonnasse Y Bockaert J Loubatieres-Mariani MM. Comparative effects of PACAP and VIP on pancreatic endocrine secretions and vascular resistance in rat. Br J Pharmacol (1996) 117(4):764–70. doi: 10.1111/j.1476-5381.1996.tb15256.x PMC1909338 8646426

[25] Gozes I Furman S. Clinical endocrinology and metabolism. potential clinical applications of vasoactive intestinal peptide: A selected update. Best Pract Res Clin Endocrinol Metab (2004) 18(4):623–40. doi: 10.1016/j.beem.2004.08.006 15533779

[26] Ahren B. Autonomic regulation of islet hormone secretion–implications for health and disease. Diabetologia (2000) 43(4):393–410. doi: 10.1007/s001250051322 10819232

[27] Havel PJ Dunning BE Verchere CB Baskin DG O'Dorisio T Taborsky GJ Jr. Evidence that vasoactive intestinal polypeptide is a parasympathetic neurotransmitter in the endocrine pancreas in dogs. Regul Pept (1997) 71(3):163–70. doi: 10.1016/s0167-0115(97)01014-8 9350974

[28] Henquin JC. Regulation of insulin secretion: A matter of phase control and amplitude modulation. Diabetologia (2009) 52(5):739–51. doi: 10.1007/s00125-009-1314-y 19288076

[29] Radosavljevic T Todorovic V Sikic B. [Insulin secretion: mechanisms of regulation]. Med Pregl (2004) 57(5-6):249–53. doi: 10.2298/mpns0406249r 15503794

[30] Ahren B. Islet G protein-coupled receptors as potential targets for treatment of type 2 diabetes. Nat Rev Drug Discovery (2009) 8(5):369–85. doi: 10.1038/nrd2782 19365392

[31] Laburthe M Couvineau A Tan V. Class II G protein-coupled receptors for VIP and PACAP: structure, models of activation and pharmacology. Peptides (2007) 28(9):1631–9. doi: 10.1016/j.peptides.2007.04.026 17574305

[32] Ramos-Alvarez I Lee L Jensen RT. Cyclic AMP-dependent protein kinase a and EPAC mediate VIP and secretin stimulation of PAK4 and activation of Na(+),K(+)-ATPase in pancreatic acinar cells. Am J Physiol Gastrointest Liver Physiol (2019) 316(2):G263–G77. doi: 10.1152/ajpgi.00275.2018 PMC6397337 30520694

[33] Winzell MS Ahren B. G-Protein-coupled receptors and islet function-implications for treatment of type 2 diabetes. Pharmacol Ther (2007) 116(3):437–48. doi: 10.1016/j.pharmthera.2007.08.002 17900700

[34] Roder PV Wu B Liu Y Han W. Pancreatic regulation of glucose homeostasis. Exp Mol Med (2016) 48:e219. doi: 10.1038/emm.2016.6 26964835 PMC4892884

[35] Jamen F Puech R Bockaert J Brabet P Bertrand G. Pituitary adenylate cyclase-activating polypeptide receptors mediating insulin secretion in rodent pancreatic islets are coupled to adenylate cyclase but not to PLC. Endocrinology (2002) 143(4):1253–9. doi: 10.1210/endo.143.4.8739 11897681

[36] Straub SG Sharp GW. Mechanisms of action of VIP and PACAP in the stimulation of insulin release. Ann N Y Acad Sci (1996) 805:607–12. doi: 10.1111/j.1749-6632.1996.tb17528.x 8993448

[37] Martin B Shin YK White CM Ji S Kim W Carlson OD. Vasoactive intestinal peptide-null mice demonstrate enhanced sweet taste preference, dysglycemia, and reduced taste bud leptin receptor expression. Diabetes (2010) 59(5):1143–52. doi: 10.2337/db09-0807 PMC2857894 20150284

[38] Filipsson K Kvist-Reimer M Ahren B. The neuropeptide pituitary adenylate cyclase-activating polypeptide and islet function. Diabetes (2001) 50(9):1959–69. doi: 10.2337/diabetes.50.9.1959 11522660

[39] Kato I Suzuki Y Akabane A Yonekura H Tanaka O Kondo H. Transgenic mice overexpressing human vasoactive intestinal peptide (VIP) gene in pancreatic beta cells. Evidence for improved glucose tolerance and enhanced insulin secretion by VIP and PHM-27 *in vivo* . J Biol Chem (1994) 269(33):21223–8. doi: 10.1016/0014-5793(94)00807-8 8063743

[40] Asnicar MA Koster A Heiman ML Tinsley F Smith DP Galbreath E. Vasoactive intestinal polypeptide/pituitary adenylate cyclase-activating peptide receptor 2 deficiency in mice results in growth retardation and increased basal metabolic rate. Endocrinology (2002) 143(10):3994–4006. doi: 10.1210/en.2002-220354 12239111

[41] Sanlioglu AD Karacay B Balci MK Griffith TS Sanlioglu S. Therapeutic potential of VIP vs PACAP in diabetes. J Mol Endocrinol (2012) 49(3):R157–67. doi: 10.1530/JME-12-0156 22991228

[42] Persson-Sjogren S Forsgren S Lindstrom P. Vasoactive intestinal polypeptide and pituitary adenylate cyclase activating polypeptide: Effects on insulin release in isolated mouse islets in relation to metabolic status and age. Neuropeptides (2006) 40(4):283–90. doi: 10.1016/j.npep.2006.04.001 16797701

[43] Prentki M Nolan CJ. Islet beta cell failure in type 2 diabetes. J Clin Invest (2006) 116(7):1802–12. doi: 10.1172/JCI29103 PMC1483155 16823478

[44] Scaglia L Smith FE Bonner-Weir S. Apoptosis contributes to the involution of beta cell mass in the post partum rat pancreas. Endocrinology (1995) 136(12):5461–8. doi: 10.1210/endo.136.12.7588296 7588296

[45] Lin EE Scott-Solomon E Kuruvilla R. Peripheral innervation in the regulation of glucose homeostasis. Trends Neurosci (2021) 44(3):189–202. doi: 10.1016/j.tins.2020.10.015 33229051 PMC7904596

[46] Imai J Katagiri H Yamada T Ishigaki Y Suzuki T Kudo H. Regulation of pancreatic beta cell mass by neuronal signals from the liver. Science (2008) 322(5905):1250–4. doi: 10.1126/science.1163971 19023081

[47] Yamamoto J Imai J Izumi T Takahashi H Kawana Y Takahashi K. Neuronal signals regulate obesity induced beta-cell proliferation by FoxM1 dependent mechanism. Nat Commun (2017) 8(1):1930. doi: 10.1038/s41467-017-01869-7 29208957 PMC5717276

[48] Imai J. Regulation of compensatory beta-cell proliferation by inter-organ networks from the liver to pancreatic beta-cells. Endocr J (2018) 65(7):677–84. doi: 10.1507/endocrj.EJ18-0241 29973428

[49] Wierstra I Alves J. FOXM1, a typical proliferation-associated transcription factor. Biol Chem (2007) 388(12):1257–74. doi: 10.1515/BC.2007.159 18020943

[50] Zhang H Ackermann AM Gusarova GA Lowe D Feng X Kopsombut UG. The FoxM1 transcription factor is required to maintain pancreatic beta-cell mass. Mol Endocrinol (2006) 20(8):1853–66. doi: 10.1210/me.2006-0056 16556734

[51] Gilon P Henquin JC. Mechanisms and physiological significance of the cholinergic control of pancreatic beta-cell function. Endocr Rev (2001) 22(5):565–604. doi: 10.1210/edrv.22.5.0440 11588141

[52] Jain S Ruiz de Azua I Lu H White MF Guettier JM Wess J. Chronic activation of a designer g(q)-coupled receptor improves beta cell function. J Clin Invest (2013) 123(4):1750–62. doi: 10.1172/JCI66432 PMC3613926 23478411

[53] Adeghate E Ponery AS Pallot DJ Singh J. Distribution of vasoactive intestinal polypeptide, neuropeptide-y and substance p and their effects on insulin secretion from the *in vitro* pancreas of normal and diabetic rats. Peptides (2001) 22(1):99–107. doi: 10.1016/s0196-9781(00)00361-2 11179603

[54] He Y Fu Q Sun M Qian Y Liang Y Zhang J. Phosphoproteome reveals molecular mechanisms of aberrant rhythm in neurotransmitter-mediated islet hormone secretion in diabetic mice. Clin Transl Med (2022) 12(6):e890. doi: 10.1002/ctm2.890 35758323 PMC9235066

[55] Thorens B. Neural regulation of pancreatic islet cell mass and function. Diabetes Obes Metab (2014) 16 Suppl 1:87–95. doi: 10.1111/dom.12346 25200301

[56] Foster N. Editorial: vasoactive intestinal peptide (vip): historic perspective and future potential. Endocr Metab Immune Disord Drug Targets (2012) 12(4):303–7. doi: 10.2174/187153012803832521 23094826

[57] Du BH Eng J Hulmes JD Chang M Pan YC Yalow RS. Guinea Pig has a unique mammalian VIP. Biochem Biophys Res Commun (1985) 128(3):1093–8. doi: 10.1016/0006-291x(85)91052-6 4004849

[58] McFarlin DR Lehn DA Moran SM MacDonald MJ Epstein ML. Sequence of a cDNA encoding chicken vasoactive intestinal peptide (VIP). Gene (1995) 154(2):211–3. doi: 10.1016/0378-1119(94)00856-n 7890166

[59] Chartrel N Wang Y Fournier A Vaudry H Conlon JM. Frog vasoactive intestinal polypeptide and galanin: primary structures and effects on pituitary adenylate cyclase. Endocrinology (1995) 136(7):3079–86. doi: 10.1210/endo.136.7.7540547 7540547

[60] Cardoso JC Vieira FA Gomes AS Power DM. The serendipitous origin of chordate secretin peptide family members. BMC Evol Biol (2010) 10:135. doi: 10.1186/1471-2148-10-135 20459630 PMC2880984

[61] Lambeir AM Durinx C Proost P Van Damme J Scharpe S De Meester I. Kinetic study of the processing by dipeptidyl-peptidase IV/CD26 of neuropeptides involved in pancreatic insulin secretion. FEBS Lett (2001) 507(3):327–30. doi: 10.1016/s0014-5793(01)02982-9 11696365

[62] Tsutsumi M Claus TH Liang Y Li Y Yang L Zhu J. A potent and highly selective VPAC2 agonist enhances glucose-induced insulin release and glucose disposal: A potential therapy for type 2 diabetes. Diabetes (2002) 51(5):1453–60. doi: 10.2337/diabetes.51.5.1453 11978642

[63] Zhu L Tamvakopoulos C Xie D Dragovic J Shen X Fenyk-Melody JE. The role of dipeptidyl peptidase IV in the cleavage of glucagon family peptides: *in vivo* metabolism of pituitary adenylate cyclase activating polypeptide-(1-38). J Biol Chem (2003) 278(25):22418–23. doi: 10.1074/jbc.M212355200 12690116

[64] Ma Y Ma M Dai Y Hong A. Expression, identification and biological effects of a novel VPAC2-specific agonist with high stability and bioactivity. Acta Biochim Biophys Sin (Shanghai) (2010) 42(1):21–9. doi: 10.1093/abbs/gmp106 20043043

[65] Ma Y Luo T Xu W Ye Z Hong A. A new recombinant pituitary adenylate cyclase-activating peptide-derived peptide efficiently promotes glucose uptake and glucose-dependent insulin secretion. Acta Biochim Biophys Sin (Shanghai) (2012) 44(11):948–56. doi: 10.1093/abbs/gms078 23052710

[66] Zhao SJ Wang DH Li YW Han L Xiao X Ma M. A novel selective VPAC2 agonist peptide-conjugated chitosan modified selenium nanoparticles with enhanced anti-type 2 diabetes synergy effects. Int J Nanomed (2017) 12:2143–60. doi: 10.2147/IJN.S130566 PMC5367579 28356733

[67] Karlsson HK Zierath JR. Insulin signaling and glucose transport in insulin resistant human skeletal muscle. Cell Biochem Biophys (2007) 48(2-3):103–13. doi: 10.1007/s12013-007-0030-9 17709880

[68] Copps KD White MF. Regulation of insulin sensitivity by serine/threonine phosphorylation of insulin receptor substrate proteins IRS1 and IRS2. Diabetologia (2012) 55(10):2565–82. doi: 10.1007/s00125-012-2644-8 PMC4011499 22869320

[69] Ishiki M Randhawa VK Poon V Jebailey L Klip A. Insulin regulates the membrane arrival, fusion, and c-terminal unmasking of glucose transporter-4 *via* distinct phosphoinositides. J Biol Chem (2005) 280(31):28792–802. doi: 10.1074/jbc.M500501200 15955810

[70] Harris JM Martin NE Modi M. Pegylation: a novel process for modifying pharmacokinetics. Clin Pharmacokinet (2001) 40(7):539–51. doi: 10.2165/00003088-200140070-00005 11510630

[71] Milla P Dosio F Cattel L. PEGylation of proteins and liposomes: a powerful and flexible strategy to improve the drug delivery. Curr Drug Metab (2012) 13(1):105–19. doi: 10.2174/138920012798356934 21892917

[72] Pan CQ Li F Tom I Wang W Dumas M Froland W. Engineering novel VPAC2-selective agonists with improved stability and glucose-lowering activity in vivo. J Pharmacol Exp Ther (2007) 320(2):900–6. doi: 10.1124/jpet.106.112276 17110523

[73] Yung SL Dela Cruz F Hamren S Zhu J Tsutsumi M Bloom JW. Generation of highly selective VPAC2 receptor agonists by high throughput mutagenesis of vasoactive intestinal peptide and pituitary adenylate cyclase-activating peptide. J Biol Chem (2003) 278(12):10273–81. doi: 10.1074/jbc.M211945200 12525492

[74] Patra JK Das G Fraceto LF Campos EVR Rodriguez-Torres MDP Acosta-Torres LS. Nano based drug delivery systems: recent developments and future prospects. J Nanobiotechnol (2018) 16(1):71. doi: 10.1186/s12951-018-0392-8 PMC6145203 30231877

[75] Klippstein R Pozo D. Vasoactive intestinal peptide (VIP) nanoparticles for diagnostics and for controlled and targeted drug delivery. Adv Protein Chem Struct Biol (2015) 98:145–68. doi: 10.1016/bs.apcsb.2014.11.006 25819279

[76] Krejs GJ. Effect of vasoactive intestinal peptide in man. Ann N Y Acad Sci (1988) 527:501–7. doi: 10.1111/j.1749-6632.1988.tb27003.x 2839086

[77] Burian B Ortner A Prassl R Zimmer A Mosgoeller W. Clinical potential of VIP by modified pharmaco-kinetics and delivery mechanisms. Endocr Metab Immune Disord Drug Targets (2012) 12(4):344–50. doi: 10.2174/187153012803832594 23094831

[78] Guan B Yan R Li R Zhang X. Selenium as a pleiotropic agent for medical discovery and drug delivery. Int J Nanomed (2018) 13:7473–90. doi: 10.2147/IJN.S181343 PMC6241719 30532534

[79] Ahmed HH Abd El-Maksoud MD Abdel Moneim AE Aglan HA. Pre-clinical study for the antidiabetic potential of selenium nanoparticles. Biol Trace Elem Res (2017) 177(2):267–80. doi: 10.1007/s12011-016-0876-z 27785741

[80] Hosnedlova B Kepinska M Skalickova S Fernandez C Ruttkay-Nedecky B Peng Q. Nano-selenium and its nanomedicine applications: a critical review. Int J Nanomed (2018) 13:2107–28. doi: 10.2147/IJN.S157541 PMC5901133 29692609

[81] Zhai X Zhang C Zhao G Stoll S Ren F Leng X. Antioxidant capacities of the selenium nanoparticles stabilized by chitosan. J Nanobiotechnol (2017) 15(1):4. doi: 10.1186/s12951-016-0243-4 PMC5217424 28056992

[82] Park JH Saravanakumar G Kim K Kwon IC. Targeted delivery of low molecular drugs using chitosan and its derivatives. Adv Drug Delivery Rev (2010) 62(1):28–41. doi: 10.1016/j.addr.2009.10.003 19874862

[83] Rao L Ma Y Zhuang M Luo T Wang Y Hong A. Chitosan-decorated selenium nanoparticles as protein carriers to improve the *in vivo* half-life of the peptide therapeutic BAY 55-9837 for type 2 diabetes mellitus. Int J Nanomed (2014) 9:4819–28. doi: 10.2147/IJN.S67871 PMC4207575 25378923

[84] Luan X Sansanaphongpricha K Myers I Chen H Yuan H Sun D. Engineering exosomes as refined biological nanoplatforms for drug delivery. Acta Pharmacol Sin (2017) 38(6):754–63. doi: 10.1038/aps.2017.12 PMC5520184 28392567

[85] Batrakova EV Kim MS. Using exosomes, naturally-equipped nanocarriers, for drug delivery. J Control Release (2015) 219:396–405. doi: 10.1016/j.jconrel.2015.07.030 26241750 PMC4656109

[86] Kim H Kim EH Kwak G Chi SG Kim SH Yang Y. Exosomes: Cell-derived nanoplatforms for the delivery of cancer therapeutics. Int J Mol Sci (2020) 22(1):14. doi: 10.3390/ijms22010014 PMC7792591 33374978

[87] Wahajuddin AS. Superparamagnetic iron oxide nanoparticles: magnetic nanoplatforms as drug carriers. Int J Nanomed (2012) 7:3445–71. doi: 10.2147/IJN.S30320 PMC3405876 22848170

[88] Zhuang M Du D Pu L Song H Deng M Long Q. SPION-decorated exosome delivered BAY55-9837 targeting the pancreas through magnetism to improve the blood GLC response. Small (2019) 15(52):e1903135. doi: 10.1002/smll.201903135 31774631

